# Supplementary figures and images for: The non-muscle actinopathy-associated mutation E334Q in cytoskeletal γ-actin perturbs interaction of actin filaments with myosin and ADF/cofilin family proteins
Source: eLife. 2024 Mar 6;12:RP93013. doi: 10.7554/eLife.93013 (PMC10942649; doi:10.7554/eLife.93013)

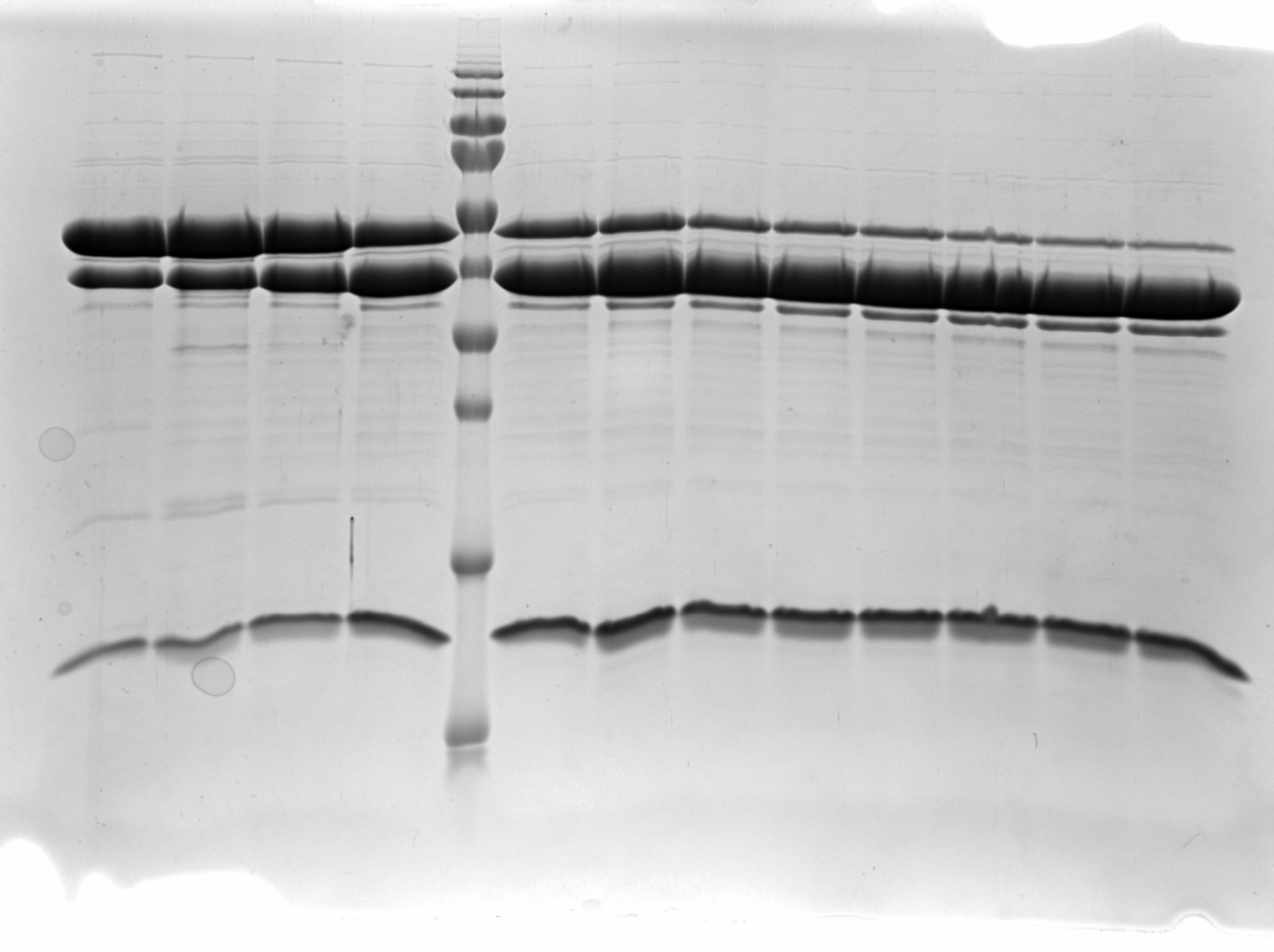

Supplement: Figure 1—figure supplement 1—source data 1. [file elife-93013-fig1-figsupp1-data1.zip › Figure1-figure supplement1-source data1.png]

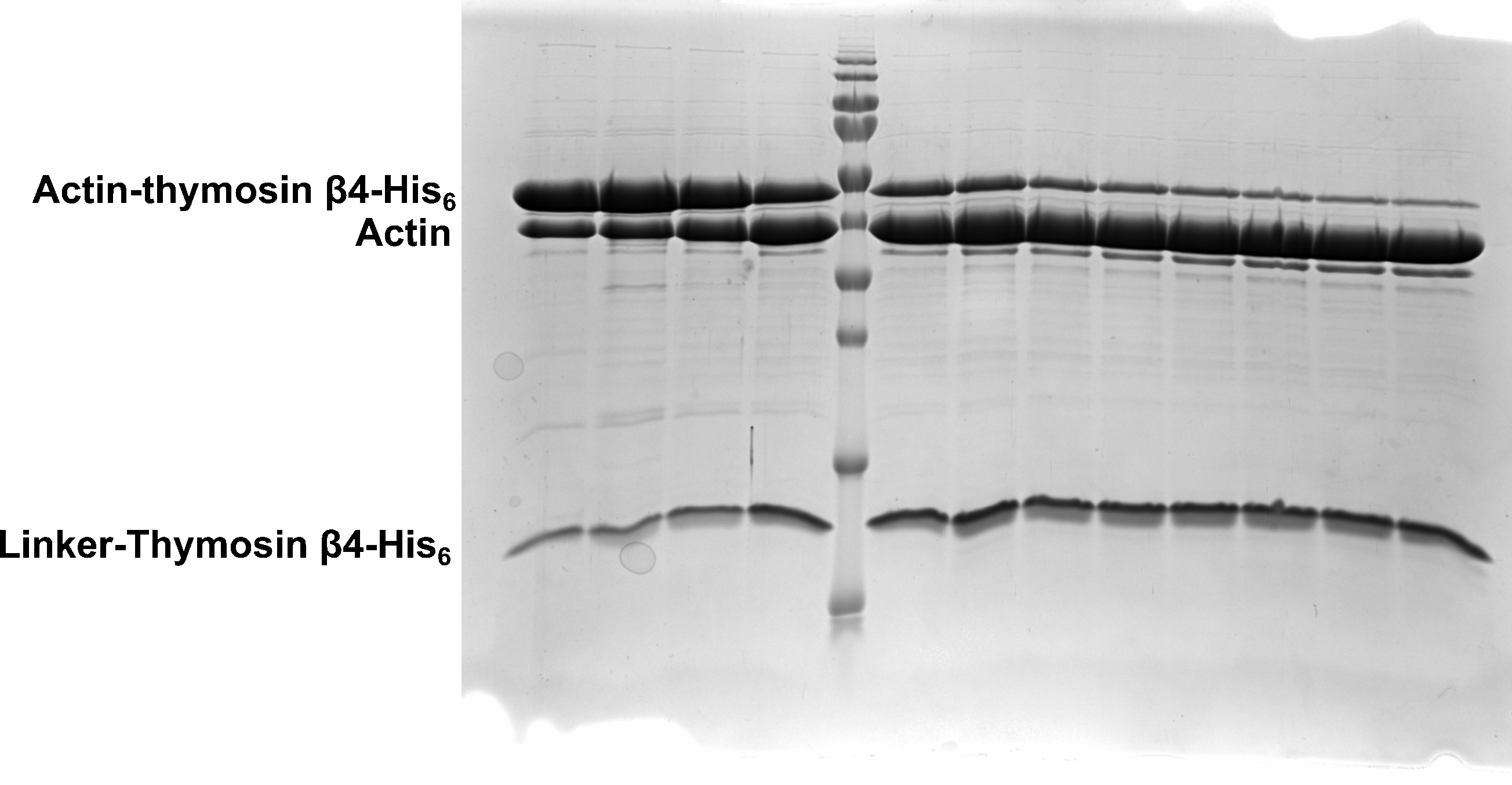

Supplement: Figure 1—figure supplement 1—source data 1. [file elife-93013-fig1-figsupp1-data1.zip › Figure1-figure supplement1-source data1_labeled.png]

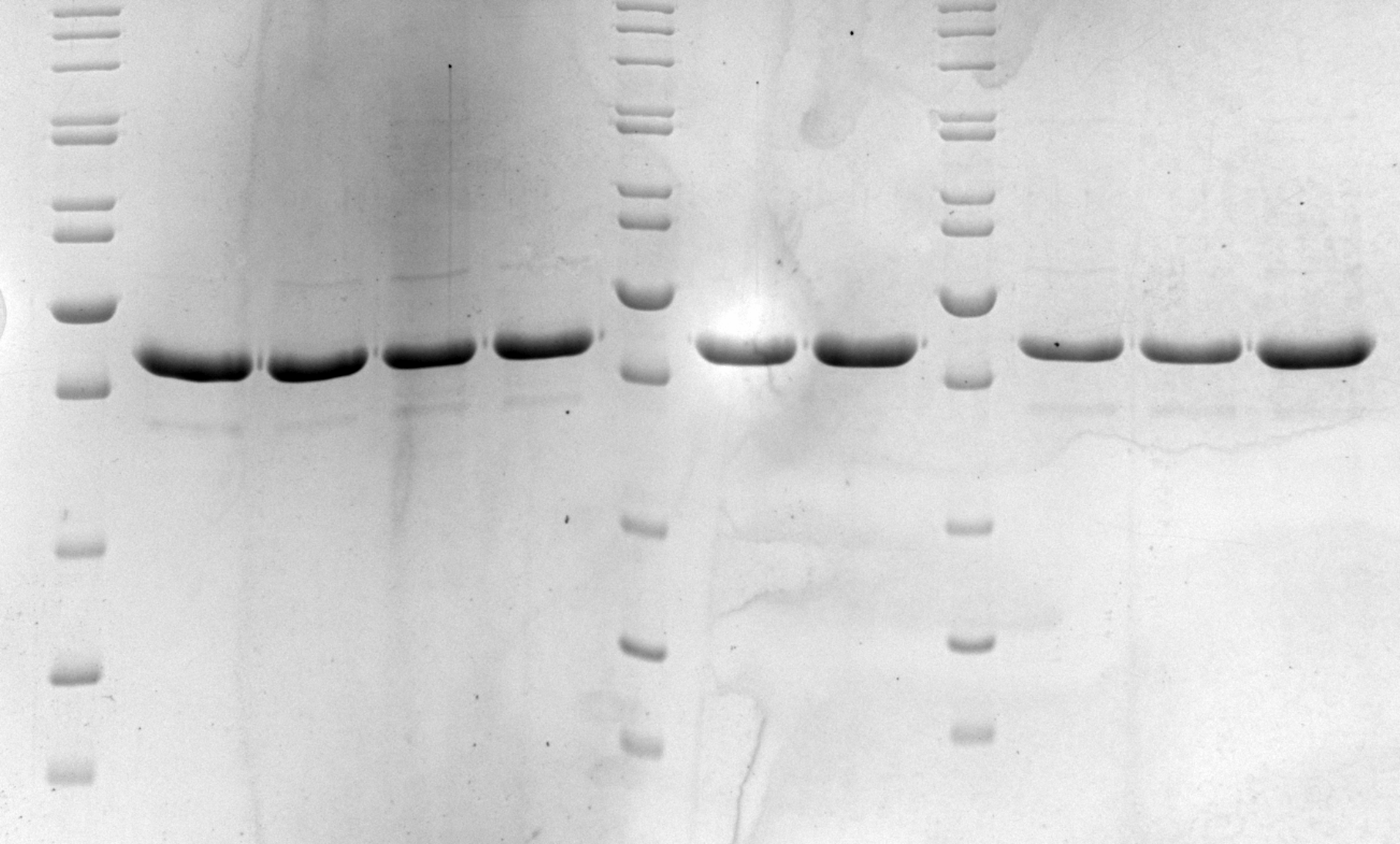

Supplement: Figure 1—figure supplement 1—source data 2. [file elife-93013-fig1-figsupp1-data2.zip › Figure1-figure supplement1-source data2.png]

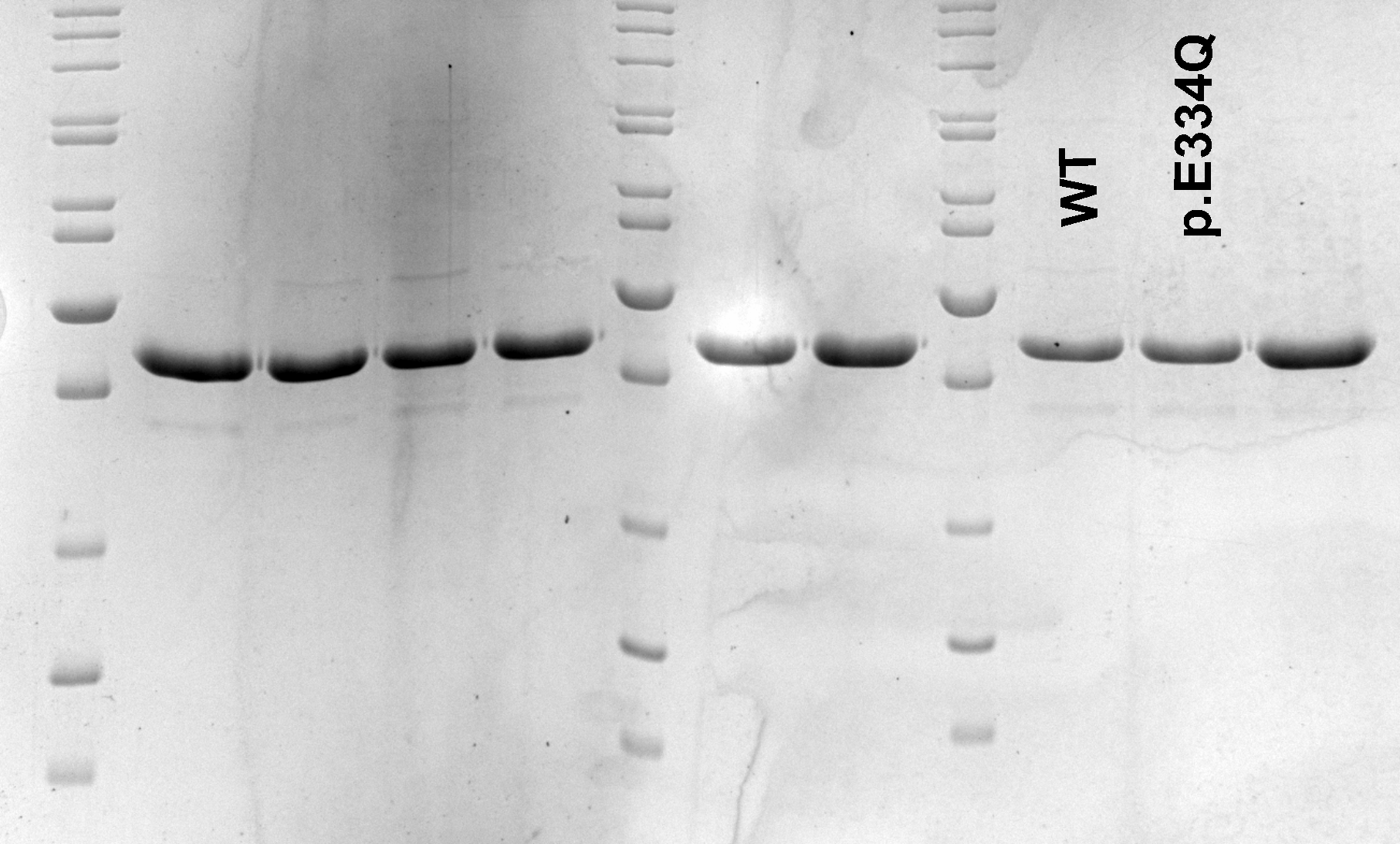

Supplement: Figure 1—figure supplement 1—source data 2. [file elife-93013-fig1-figsupp1-data2.zip › Figure1-figure supplement1-source data2_labeled.png]

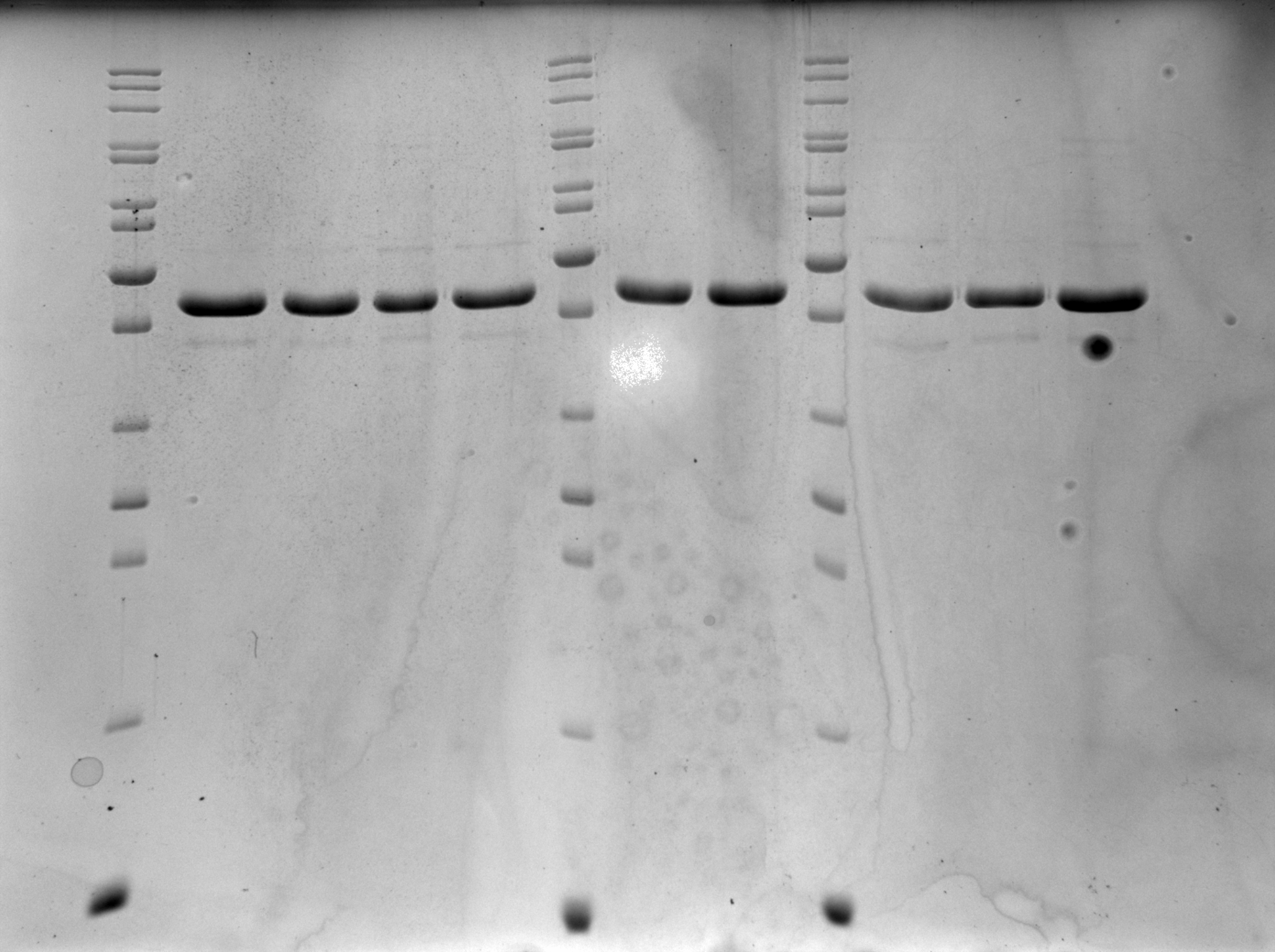

Supplement: Figure 1—figure supplement 1—source data 3. [file elife-93013-fig1-figsupp1-data3.zip › Figure1-figure supplement1-source data3.png]

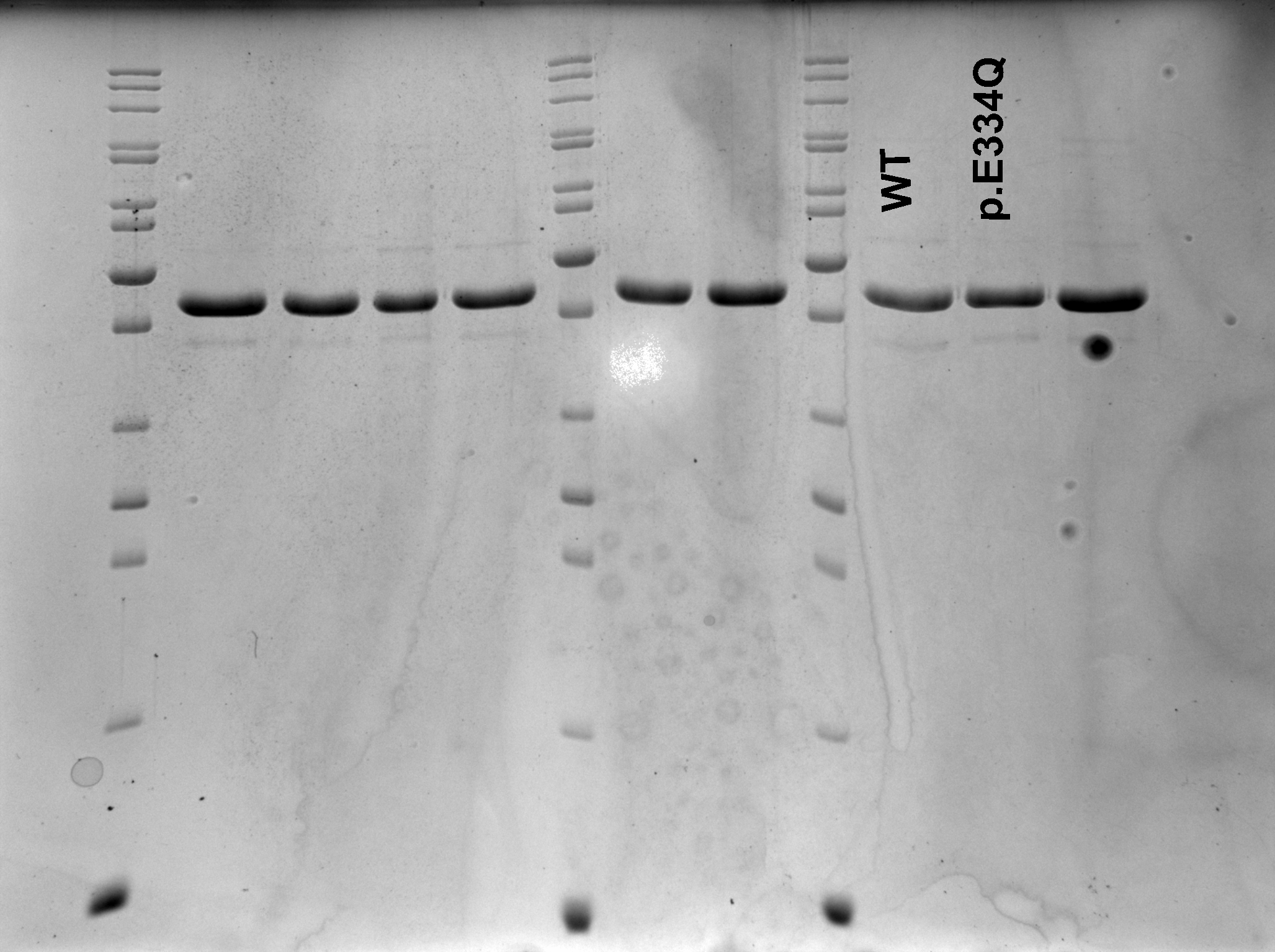

Supplement: Figure 1—figure supplement 1—source data 3. [file elife-93013-fig1-figsupp1-data3.zip › Figure1-figure supplement1-source data3_labeled.png]

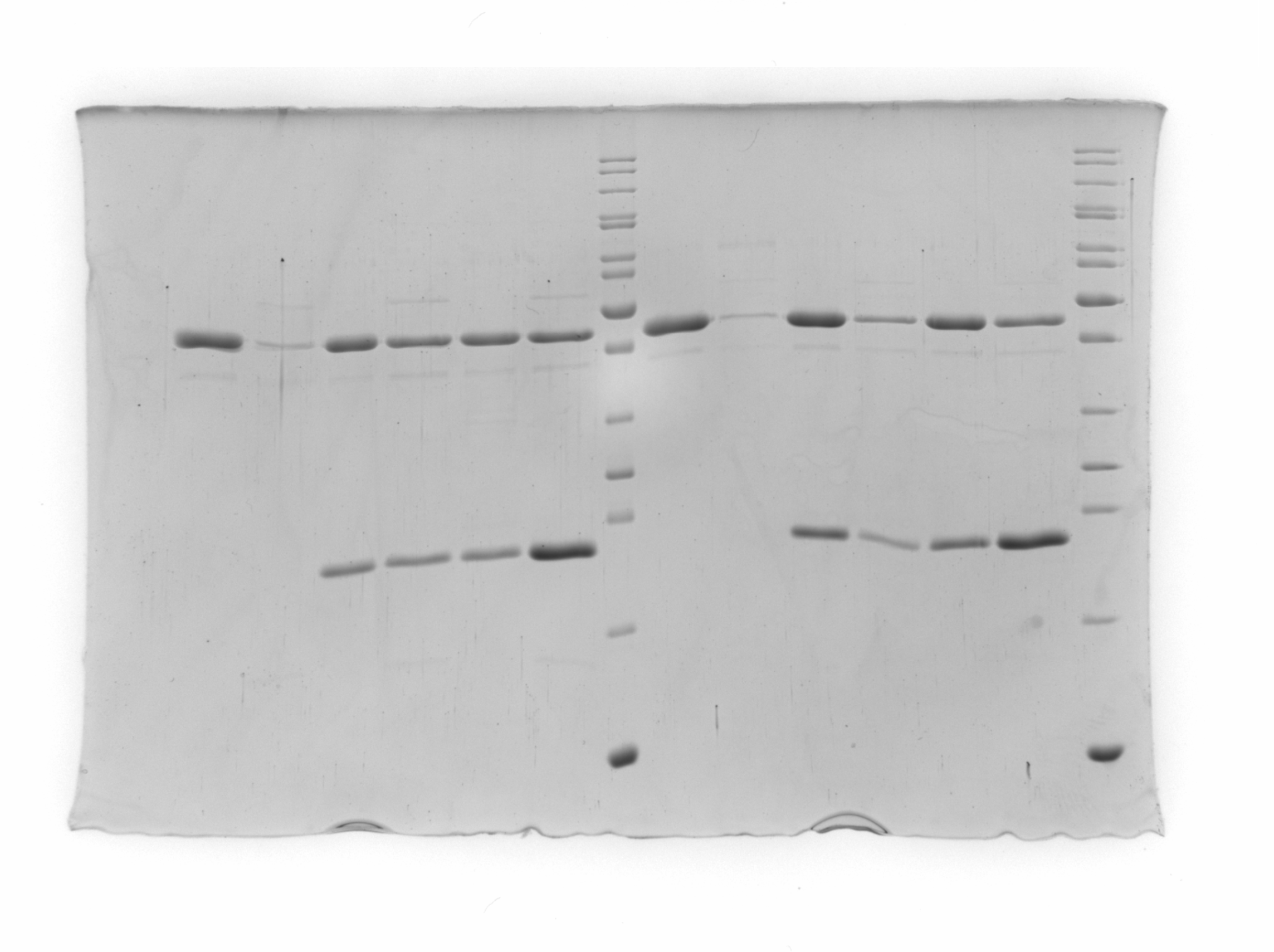

Supplement: Figure 5—figure supplement 2—source data 1. [file elife-93013-fig5-figsupp2-data1.zip › Figure5-figure supplement2-source data1.png]

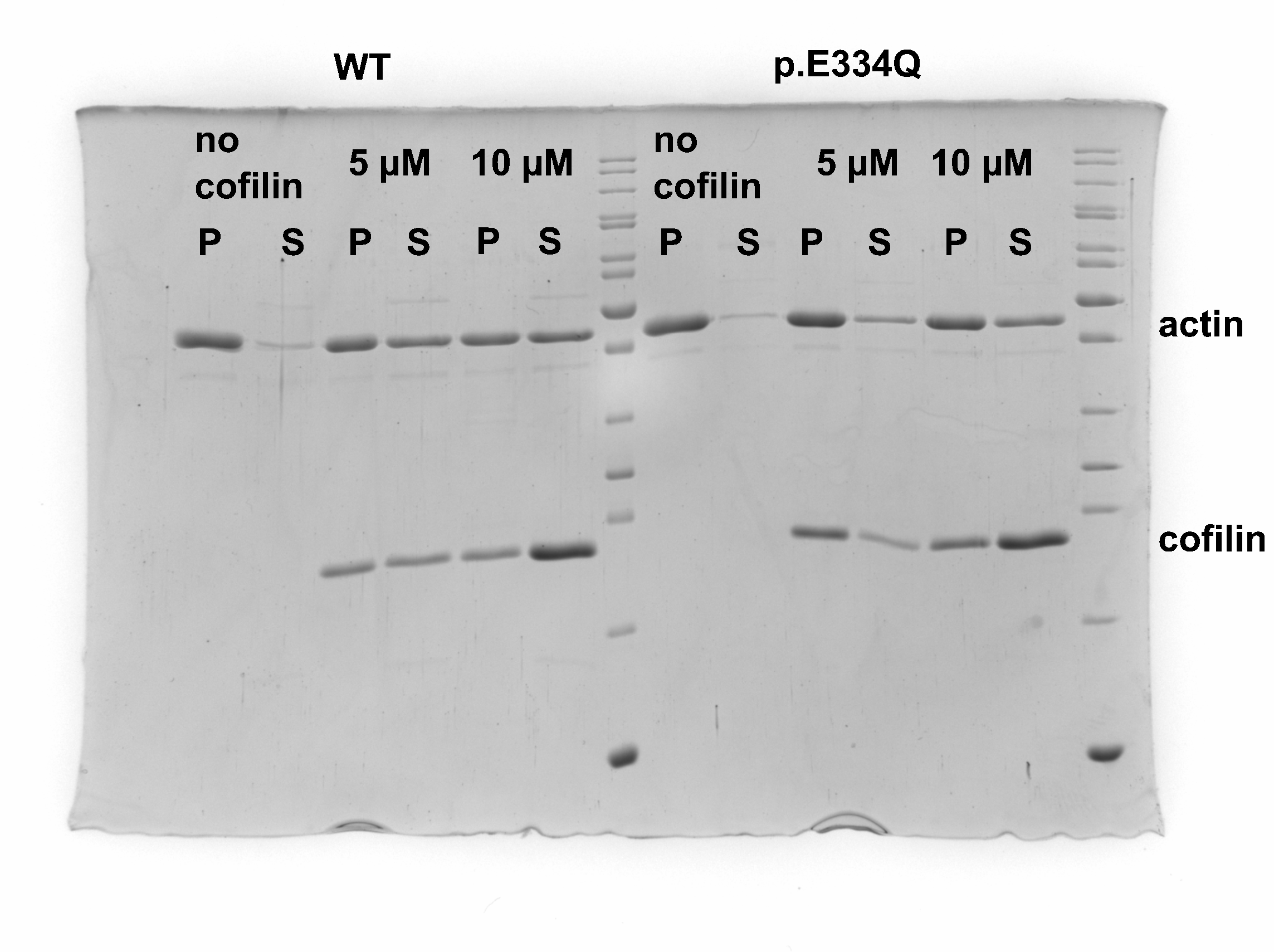

Supplement: Figure 5—figure supplement 2—source data 1. [file elife-93013-fig5-figsupp2-data1.zip › Figure5-figure supplement2-source data1_labeled.png]
